# Where and How Wolves (*Canis lupus*) Kill Beavers (*Castor canadensis*)

**DOI:** 10.1371/journal.pone.0165537

**Published:** 2016-12-19

**Authors:** Thomas D. Gable, Steve K. Windels, John G. Bruggink, Austin T. Homkes

**Affiliations:** 1 Department of Biology, Northern Michigan University, Marquette, Michigan, United States of America; 2 Voyageurs National Park, International Falls, Minnesota, United States of America; University of Alberta, CANADA

## Abstract

Beavers (*Castor canadensis*) can be a significant prey item for wolves (*Canis lupus*) in boreal ecosystems due to their abundance and vulnerability on land. How wolves hunt beavers in these systems is largely unknown, however, because observing predation is challenging. We inferred how wolves hunt beavers by identifying kill sites using clusters of locations from GPS-collared wolves in Voyageurs National Park, Minnesota. We identified 22 sites where wolves from 4 different packs killed beavers. We classified these kill sites into 8 categories based on the beaver-habitat type near which each kill occurred. Seasonal variation existed in types of kill sites as 7 of 12 (58%) kills in the spring occurred at sites below dams and on shorelines, and 8 of 10 (80%) kills in the fall occurred near feeding trails and canals. From these kill sites we deduced that the typical hunting strategy has 3 components: 1) waiting near areas of high beaver use (e.g., feeding trails) until a beaver comes near shore or ashore, 2) using vegetation, the dam, or other habitat features for concealment, and 3) immediately attacking the beaver, or ambushing the beaver by cutting off access to water. By identifying kill sites and inferring hunting behavior we have provided the most complete description available of how and where wolves hunt and kill beavers.

## Introduction

Wolves (*Canis lupus*) primarily prey upon large ungulate species [1]. However, they are opportunistic hunters and use alternative prey species seasonally when they are abundant, vulnerable, and easy to capture [2–5]. Wolves and beavers co-occur throughout the boreal ecosystem, and wolves can be significant predators of beavers [6,7]. During winter, beavers are usually in their lodges or foraging below the ice and thus are seldom available to wolves [8]. From ice-out in spring through freeze-up in late fall beavers must forage on land to increase fat reserves and to re-supply food caches to survive the upcoming winter [9,10]. Consequently, wolf predation of beavers is highest during this period of vulnerability, and beavers can be important prey for wolves [11–13]. Indeed, wolves have used beaver as a secondary or tertiary prey item in many areas [2,14–17]. In some systems under certain conditions, such as high beaver densities or low ungulate densities, beavers can actually be the primary summer prey of wolves [6,13,18].

Despite this, little is known about wolf-beaver interactions in systems where the species co-occur. In particular, the manner in which wolves hunt, attack, and capture beavers is unknown. In a comprehensive review of wolf hunting behavior, Mech et al. [19] concluded that there were “no actual descriptions of wolves hunting beavers”. The lack of observations is not surprising as riparian vegetation is often dense around active beaver habitats during the ice-free season, and in winter beavers spend most of their time below the ice where they are safe. Thus, other methods must be used to understand how wolves hunt beavers.

A common method to understand wolf predation on ungulates is to document kill sites by searching areas where there were clusters of locations from GPS-collared wolves [20–22]. However, finding kill sites of small prey species is difficult because wolves can consume the entire carcass in a short period [23–25]. Nonetheless, some studies have successfully documented beaver kill sites at clusters [20, 21, 25, 26]. Thus, we sought to infer wolf hunting behavior from beaver kill sites to understand how and where wolves hunt beavers.

## Materials and Methods

### Study Area

**Fig 1:**
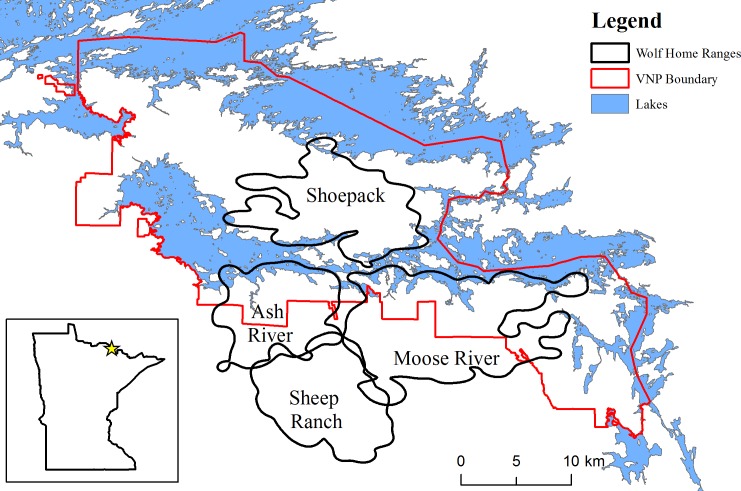
The 95% adaptive kernel home ranges of the 4 wolf packs studied in and around Voyageurs National Park (VNP), Minnesota, USA in 2015.

Voyageurs National Park (VNP) is located in northern Minnesota (USA) along the Ontario (Canada) border (48°30' N, 93°00' W). Voyageurs National Park is an 882 km^2^ landscape dominated by forests and lakes, with nearly 50% of the park composed of aquatic habitat types (Fig 1) [27]. Four large lakes cover 342 km^2^ (39%) of the park, and 26 smaller lakes are scattered throughout the landmasses of the park. Beaver impoundments are abundant as the park has sustained high beaver densities (>1 active colony/km^2^) for over 40 yr [28,29]. Voyageurs National Park is in the Laurentian Mixed Forest Province, which is a transition zone between the southern boreal forest and northern hardwood forest [30]. As a result, the park is a mosaic of deciduous and coniferous forests. Typically, lakes freeze during late October to mid-November with ice-out occurring during late April to early May [31].

White-tailed deer (*Odocoileus virginianus*) are common in VNP while moose (*Alces americanus*) are relatively rare [32]. Wolf densities in the area are high (4–6 wolves/100 km^2^), and the average summer home-range size in 2015 was 115.8 km^2^ (VNP, unpublished data). Hunting and trapping are not allowed in the park. Recreational trapping of beavers outside the park is common. Wolf hunting and trapping are illegal in Minnesota at present but are legal in Ontario.

### Wolf Capture and Collaring

As part of a broader wolf monitoring program, we captured wolves from packs within or near the park during 2012–2015 using #7 EZ Grip foothold traps (Livestock Protection Company, Alpine, Texas). We set 10–30 traps in each pack’s territory for approximately 2 weeks or until we captured our target number of wolves. We checked traps at least once/day. We immobilized captured wolves with 10 mg/kg ketamine and 2 mg/kg xylazine using a syringe pole. Once immobilized, we fitted wolves with global positioning system (GPS) telemetry collars (Lotek IridiumTrackM 1D or 2D, Lotek Wireless Inc, Newmarket, Ontario, Canada; Vectronic Vertex Survey, Vectronic Aerospace, Berlin, Germany). Morphological measurements, tissue samples, and blood were collected. Sex and age were also recorded. We reversed wolves with 0.15 mg/kg of yohimbine and monitored through recovery. GPS-collar fix intervals were set to 20 minutes, 4 hours, 6 hours or 12 hours, depending on the collar type, where the pack was located, and whether there was > 1 collar in the pack at that time.

We used data from 6 collared wolves from 4 different packs in this study. In 2014, we deployed a 12-hr-fix-interval collar on a 2-yr-old non-breeding female from the Sheep Ranch Pack. We recaptured this individual in August 2015 and replaced the collar with a 20-min-fix-interval collar. We deployed 2 6-hr-fix-interval collars in 2014 (fix schedules were changed to 4 hr in May 2015) on a breeding male (about 5 yr old in 2015) and a non-breeding female (about 3 yr old in 2015) from the Moose River Pack. We also deployed a 20-min-fix-interval collar in June 2015 on a 2-yr-old non-breeding male from the Moose River Pack. We deployed a 4-hr-fix-interval collar (fix schedule was changed to 6-hr in May 2015) in 2013 on a breeding male (about 6 yr old in 2015) from the Ash River Pack and a 20-min-fix-interval collar on a 2-yr-old non-breeding female from the Shoepack Lake Pack in September 2015.

Locations were uploaded (12 locations/upload) every 4 hours to 6 days depending on the fix interval. All capture and handling of wolves was approved by the National Park Service’s Institutional Animal Care and Use Committee (MWR_VOYA_WINDELS_WOLF) and conducted in accordance with American Society of Mammalogists Guidelines for use and handling of wildlife mammals for research [33].

### Clusters and Identifying Kill Sites

From April 2015 to November 2015 we examined localized clusters of wolf activity to document kill sites. Potential kill sites were determined by identifying clusters of locations from GPS-collared wolves using ArcGIS 10.2 [34]. Clusters were defined as consecutive locations within 200 m for ≥4 hours [35]. We examined clusters 1–28 days ($\bar{x}$ = 10 ± 8 days) after the wolf or wolves were present.

Once at clusters we systematically searched an area with a 100-m radius around the cluster for evidence of a kill site. Searched clusters were classified as either kill sites, bed sites, or unknown. We also recorded when clusters were in active beaver habitats. We considered clusters to be in active beaver habitats if ≥50% of cluster locations were within 15 m of an active beaver habitat feature (e.g. feeding trail, dam, feeding canal, shoreline of active pond, etc.), or if we found a beaver kill site. Because we were primarily focused on identifying beaver kill sites, the clusters we searched were not representative of all the clusters from collared wolves during our study. However, all clusters searched in active beaver habitats should be representative of all clusters in active beaver habitats as we visited those clusters based only on the accessibility of the cluster.

When we identified kill sites, we searched an area with a 200-m radius around the kill site to identify all prey remains and wolf sign present. We also collected prey remains, noted the location of the kill, the presence of other predators/scavengers, and photographed the site. We recorded all wolf and beaver sign at kill sites as well as evidence of a struggle such as drag marks, depressed vegetation, and blood trails. At opportunistically-found kill sites (i.e., those encountered without the assistance of GPS-collar clusters), we examined the area thoroughly for evidence (tracks, scats, beds, hair) of predators. If only wolf sign was present, we considered the prey to have been killed by wolves.

**Fig 2:**
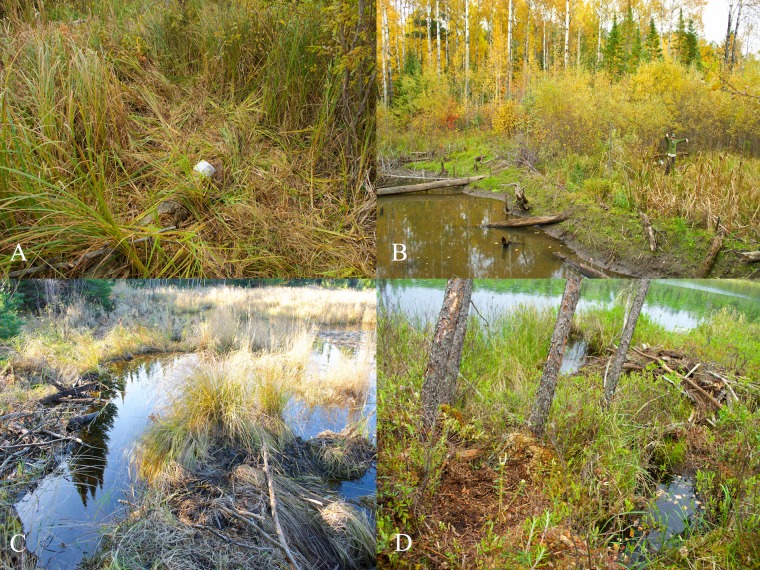
**Examples of evidence found at beaver kill sites (A,B,C), and of wolf behavior when in active beaver habitats (D) in Voyageurs National Park 2015.** A) Matted vegetation at kill sites provided important information about how wolves killed beavers. B) Co-author Homkes stands at Beaver Kill Site 13 <10 m below an active beaver dam. C) Beaver Kill Site 18 on a small point <5 m from the active dam where a wolf, based on the trampled vegetation, presumably pulled a kit beaver out of the water and consumed it. D) A wolf bed (lower left corner) found when examining clusters of GPS-locations in the spring. The wolf bedded for ≥4 hr next to this active beaver lodge without making a kill.

Kill sites were typified by a disturbed area with beaver remains such as fur, stomach contents, bone fragments, castor glands, and skull remnants (Fig 2). We were able to differentiate between kill sites and feeding sites because kill sites were usually close to water and there was significant disturbance around the area whereas feeding sites were usually wolf beds (with prey remains present) that were further inland than the actual kill site. We estimated carcass utilization to the nearest 5%, with 99% representing the greatest carcass utilization still detectable. We estimated the distance of the kill site to the nearest body of water (lake, pond, river, or stream) by examining May 2015 aerial imagery in Google Earth Pro 7.1.4 [36].

Collared wolves were estimated to be alone at kill sites if: 1) all beaver remains found were at the site or at GPS locations, and 2) there was only 1 wolf bed at the site, or all wolf beds at the site were associated with GPS locations [25]. We determined the minimum time a collared wolf was at a kill site based on the time between the first and last location at the site. Maximum time spent at a kill site was determined by taking into account the fix interval prior to and after the first and last locations respectively (e.g., if minimum time spent was 8 hr and the fix interval was 4 hr, then the maximum time spent at the kill site was 16 hr). Due to the large fix intervals, these numbers provide the range of time wolves spent at kill sites. Thus we calculated the estimated time spent at kill sites as the means of the minimum and maximum times spent at kill sites.

## Results

We identified 22 beaver kill sites from 2 April 2015 to 10 November 2015. Of these, 12 were in spring (2 April– 29 May) and 10 in fall (20 September– 4 November). We found 4 kill sites from GPS collars with 20-min fix intervals, 7 from GPS collars with 4-hr fix intervals, 8 from GPS collars with 6-hr fix intervals, 1 from GPS collars with 12-hr fix intervals, and 2 kill sites were found opportunistically (Table 1). Collar fix success for 4/6-hr collars (combined because collars were the same but fix schedule was changed during study) was 81.8% with a total of 1,966 fixes recorded. For 20-min collars fix success was 99.0% and for 12-hr collars fix success was 74.8% with a total of 20,423 and 101 fixes recorded, respectively.

**Table 1:** Characteristics of beaver kill sites from GPS-collared wolves in Voyageurs National Park in 2015.

Kill Site No.	Date of Kill	Season	Wolf ID	Pack	GPS Collar Fix Interval	Kill Site Type	Time Spent (hr)^a^	Distance from Water (m)
1	4/14/15	Spring	V009	Ash River	4 hr	Below Dams	24.0	15
2	4/12/15	Spring	V009	Ash River	4 hr	Below Dams	12.0	11
3	4/8/15	Spring	V009	Ash River	4 hr	Near Shores	16.0	27
4	UNK	Spring	V009	Ash River	N/A^b^	Near Shores	UNK^b^	16
5	4/2/15	Spring	V009	Ash River	4 hr	At Lodges	12.0	15
6	4/27/15	Spring	V028	Moose River	6 hr	Near Shores	12.0	16
7	4/27/15	Spring	V027	Moose River	6 hr	Feeding Trails	18.0	18
8	5/3/15	Spring	V009	Ash River	4 hr	Feeding Trails	13.0	99
9	5/8/15	Spring	V009	Ash River	6 hr	Below Dams	12.0	1
10	4/27/15	Spring	V009	Ash River	4 hr	Small Waterways	8.0	8
11	5/29/15	Spring	V027	Moose River	4 hr	Small Waterways	4.1	1
12	5/20/15	Spring	V026	Sheep Ranch	12 hr	Forest Interior	24.0	222
13	9/20/15	Fall	V009	Ash River	6 hr	Below Dams	36.0	4
14	10/13/15	Fall	V009	Ash River	6 hr^c^	Feeding Trails	6.1	16
15	9/30/15	Fall	V009	Ash River	6 hr	Feeding Canals	12.0	6
16	10/11/15	Fall	V009	Ash River	6 hr	Feeding Canals	36.0	3
17	10/19/15	Fall	V009	Ash River	6 hr	Feeding Trails	18.0	5
18	10/17/25	Fall	V045	Shoepack	20 min	At Dams	8.0	1
19	10/20/15	Fall	V026	Sheep Ranch	20 min	Feeding Trails	7.9	9
20	10/29/15	Fall	V045	Shoepack	20 min	Feeding Trails	26.4	23
21	10/28/15	Fall	V009	Ash River	6 hr	Feeding Trails	12.0	13
22	11/5/15	Fall	V033	Moose River	20 min	Feeding Trails	5.7	13

^a^Mean estimated time at kill site calculated by taking the mean of the minimum and maximum time spent at a kill site.

^b^Not applicable because kill site was identified opportunistically and not from collared wolves.

^c^Discovered opportunistically but the collared wolf (6 hr fixes) was <100 m from the kill site 2 hr after we found it. The fresh blood indicated that the kill was <6 hr old when discovered because the blood would have been washed away by rain earlier that morning.

We searched a total of 142 clusters of which 54 were in active beaver habitats. Of the 43 kill sites we identified at clusters, 22 were beaver kill sites, 19 were deer (14 adults/yearlings and 5 fawns), 1 was a great blue heron (*Ardea herodias*), and 1 was a snowshoe hare (*Lepus americanus*). Of the clusters in active beaver habitats, 20 (37%) were beaver kill sites and 34 (63%) were wolf bed sites. We found either a bed site or kill site at all clusters in active beaver habitats and thus did not classify any clusters in active beaver habitats as unknown. We concluded that collared wolves were alone at 16 (73%) kill sites, with other wolves at 4 (18%) sites, and are uncertain about the remaining 2 sites.

Generally, beaver kill sites were difficult to detect as mean carcass utilization was 98% (range: 85–100%). We were able to recover the lower mandible, skull, or teeth at 7 (32%) kill sites. At most of the kill sites we visited, all remains found were located where the beaver appeared to have been killed. However, at some sites remains (often the skull) were also found up to 180 m away. We did not document any sign of other predators or scavengers at any of the kill sites we visited. The mean minimum time wolves spent at kill sites was 10.6 ± 8.0 hr (range: 4.0–30.0), the mean estimated time spent, 15.4 ± 9.2 hr (range: 5.7–36.0), and the mean maximum time spent, 20.2 ± 11.1 hr (range: 6.0–48.0).

Kill sites ranged from 1 to 222 m ($\bar{x}$ = 24.5 ± 48.5 m) from water, and all but 2 sites were <27 m from water. With the 2 farthest distances (99 m and 222 m) excluded, the mean distance from water was 10.9 ± 7.5 m. Based on depressed vegetation and drag trails from water to kill sites, wolves appeared to have attacked beavers in the water and pulled them out at 6 (27%) kill sites. Five of these kill sites were <5 m from water. Although we did find drag marks at kill sites from the wolf killing and consuming the beaver we found no evidence to suggest wolves killed beavers and then carried the carcass to a different location to consume it.

We classified kill sites into 8 categories based on the location of the kill site and our interpretation of how wolves killed the beaver. We documented seasonal variation in kill site type and frequency. Kill sites below the dam and on shore composed 50% of all spring kill sites, whereas kill sites near feeding trails and feeding canals composed 80% of all fall kill sites. Descriptions and a map of the locations of individual kill sites can be found in S1 Appendix and S1 Map, respectively.

### At Dams

We identified 1 kill site where a wolf killed a kit beaver (based on small size of incisors found at the kill site) while on a small point 5 m from a small beaver dam (Fig 2).

### At Lodges

In spring, water levels in VNP can be >1 m lower than during the previous fall. As a result many shoreline beaver lodges are left completely out of the water in spring until water levels increase [37]. Thus, beavers must travel over land (up to 100 m) exposed to predators to reach open water. We documented 1 kill site that occurred 10 m from a lodge that had no open water nearby. Based on trampled vegetation and drag marks, it appears that the wolf caught the beaver near the lodge (drag marks with tufts of fur present started 1 m from lodge) and then dragged the beaver 10 m behind the lodge where the wolf ate the beaver.

### Below Dams

We identified 4 kill sites below beaver dams. In 2 instances beavers were in the small channels below the dam when, based on the matted vegetation, they appeared to have been attacked in the water, pulled out, and killed nearby. The kill sites were 28 and 33 m downstream from the dams. In the other 2 instances, the beavers were on land when attacked; these kill sites were much closer to the dams (8 and 10 m; Fig 2).

### Feeding Canals

We documented 2 kill sites where a wolf or wolves appeared to have attacked and pulled beavers out of feeding canals. In both instances, the feeding canals were at least 1 m deep and 1 m wide, and there was trampled vegetation and drag marks leading from the canals to the kill sites. The beavers were consumed <5 m from the feeding canals.

### Feeding Trails

We documented 8 kills that occurred on or near feeding trails. With the exception of 1 kill site that was 99 m from water, kill sites on feeding trails were 5.1–23.1 m from water ($\bar{x}$ = 13.3 ± 5.9 m).

### Near Shores

We documented 3 kill sites near or on the shoreline of a lake or river. These kill sites were not near any feeding trails, and there was no evidence of fresh cuttings nearby. All 3 sites were <200 m from active lodges so the beavers killed at these sites probably were not dispersing.

### Small Waterways

We identified 2 kill sites where, based on matted vegetation and drag marks, wolves appeared to have attacked and pulled beavers out of small waterways. Beavers used these waterways to travel between bodies of water and both sites were >200 m from the nearest known lodge. Although kill sites along small waterways are similar to kill sites at feeding canals, they differ in that beavers traveling in feeding canals are moving from water to land to forage whereas in small waterways beavers are traveling between bodies of water.

### Forest Interior

We documented 1 instance of a wolf killing a beaver in a dense aspen stand 222 m from the nearest body of water. We found no evidence of fresh cuttings or beaver activity near the kill site. At the kill site we found evidence of a struggle as a downed log had been torn apart on 1 end with claw and/or tooth marks present in the wood. A small sapling had also been broken off about 1 m above the ground, and we found beaver fur on the sapling where it had been broken off. Thus, we assumed that this was a dispersing beaver traveling through the woods to reach a body of water when a wolf either opportunistically encountered and killed it, or scent-tracked it from the water.

## Discussion

Fifty years ago, Mech [38] stated, “the manner in which wolves hunt beavers is unknown”. Since then thousands of hours of wolf observations have occurred across the world, and still no observations of a wolf hunting a beaver exist [19]. Although there are limitations when inferring hunting behavior from kill sites, we think that the combination of physical evidence at kill sites and how wolves spent time in active beaver habitats provide a viable substitute to visual observations of predation behavior (Fig 2).

We documented more beaver kill sites (22) than previous studies by investigating areas where clusters of locations from GPS-collared wolves occurred. We were surprised that 80% (17/22) of the beaver kill sites were identified via collars with fix intervals ≥4 hr as previous studies have discussed the challenges of identifying kill sites of small prey even with short fix intervals (≤30 min) [25,26,39]. Our success in finding these kill sites was in part a result of wolves spending relatively long periods ($\bar{x}$ = 15.4 hr) at kill sites (Table 1).

Wolves appeared to have been alone at 73% of beaver kill sites, which is to be expected as wolves frequently travel alone from spring through early fall [24,25,40]. We acknowledge that it is challenging to determine whether the wolf was alone without short fix intervals or having multiple pack members collared. However, at most of the kill sites where we inferred the wolf was alone, we only found 1 wolf bed or all the beaver remains were in a small area and there were no other wolf beds nearby, both of which suggest the wolf was alone. Beavers in VNP can exceed 20 kg and can be a substantial meal for a wolf [37]. Peterson and Ciucci [1] stated that a 20 kg beaver can be entirely consumed within a few hours, especially with multiple wolves present. Although wolves might consume a beaver quickly, our results suggest wolves can remain at beaver kill sites for a substantial period regardless of whether alone (15.6 hr) or with others (15.0 hr). However, our estimates of time spent at kill sites might be positively biased because we would not have detected kill sites where wolves were present <4 hr due to how we defined clusters. Moreover, we do not know whether characteristics of kill sites where wolves were present for ≥4 hr are different from kill sites where they are present <4 hr.

### Where Wolves Kill Beavers

During spring, wolves appear to hunt and kill beavers at or near a variety of habitat features. In fall, beavers must travel on land more frequently to access, obtain, and transport food both to store in the cache and to consume [10,41]. Therefore, it is not surprising that 80% (8/10) of kill sites in fall were at feeding canals or trails [6,8]. Mech et al. [19] postulated that wolves likely hunt beavers during the ice-free season by targeting beaver trails going inland. Our results agree with this, though this strategy appears to be much more prevalent in fall than spring. We did not identify any beaver kill sites at clusters searched during June–August. Beavers can compose a substantial portion of wolf diets during this period and further research is needed to understand where wolves kill them [15,18].

Mech and Peterson [42] and Peterson and Ciucci [1] speculated that wolves kill beavers near beaver dams based on the amount of time wolves and beavers spend near beaver dams. We confirmed this as 5 (23%) kills occurred at, or below, beaver dams. However, kill sites near dams were more prevalent in spring than fall, consistent with our observations that wolves spent a substantial period near active beaver dams in spring but not fall. We speculate that wolves might wait below dams because if a beaver was on the down slope of the dam it would be challenging for the beaver to turn around before it was attacked (see Kill Sites 2 and 13, S1 Appendix). Much of this is based on observations of clusters where wolves appeared to have bedded down <3 m from small channels or beaver trails below dams for several hours but never made a kill.

### How Wolves Hunt Beavers

We think a typical hunting strategy in our study area consists of 3 components: 1) waiting near areas of high beaver use (e.g., feeding trails) until the beaver comes near shore or ashore, 2) using vegetation, the dam, or other habitat features for concealment, and 3) attacking the beaver by cutting off access to water, or immediately attacking the beaver (e.g. ambush). If wolves were simply killing beavers opportunistically, most clusters in active beaver habitats should be kill sites because wolves would only travel by these features rather than bedding down next to them. However, of all clusters <15 m from active beaver habitat features 63% were bed sites. We think this is evidence that wolves wait near active beaver habitats as a hunting strategy to kill beavers. If wolves wait for beavers, it follows that they would have to use concealment to avoid being detected.

Others have speculated that waiting near areas of beaver use would be a profitable strategy for wolves [1,19]. Thurber and Peterson [43] observed a lone wolf that they thought was actively hunting beavers during mid-winter thaws by bedding down next to beaver trails. Wolves appear to exhibit this ambushing behavior when hunting other prey species as well [44]. Mech [45] observed wolves waiting for 3 hr in a depression to ambush muskoxen (*Ovibos moschatus*)–even though the herd was only a few hundred meters away–and concluded that it appeared that the wolves chose the location to maximize their chance of success. Compared to ungulates, beavers have small home ranges and are very predictable, as they must eventually come on shore to forage or cross over their dams to reach another body of water. Thus, waiting concealed at these areas should be an effective strategy for wolves.

Once a wolf has located a beaver on or near land we think it either attacks the beaver by cutting off access to the water, or ambushing the beaver. At kill sites 7 and 14, fresh wolf tracks indicate wolves followed the shoreline to a feeding trail, then followed the feeding trail and killed a beaver <20 m from water on that trail. Basey and Jenkins [46] thought that intercepting a beaver or cutting off its path to water was the most likely strategy for any predator hunting beavers. Similarly, Mech [47] suggested that wolves might follow shorelines until they find a beaver inland that they could easily subdue.

However, we think wolves also use ambush as a strategy to hunt beavers. At 27% (6/22) of kill sites, clear drag marks from the water to the kill site suggested wolves attacked beavers in the water and consumed them close by on shore. In such cases, wolves likely are not waiting for the beaver to move inland before attacking it (see kill sites #2, #5, #12, and #13, S1 Appendix). For this to happen, 1 of 3 scenarios must have occurred: 1) the wolf attacked the beaver on land but the beaver was able to get back to the water where it was subsequently subdued, 2) the wolf waited by the water, determined it could successfully kill the beaver, and attacked the beaver in the water, or 3) the beaver reached the water after detecting the wolf but was intercepted by the wolf in the water. In 83% (5/6) of these kills, beavers were pulled from waterways or feeding canals that were both ≥1 m deep and wide. Given the depth and width of these waterways and canals, it would seem beavers would be able to avoid being captured once they reached the water, which make scenarios 1 and 3 unlikely. Therefore, scenario 2 appears to be the most plausible explanation for how wolves attacked beavers in the water and killed them on land. However, we do not know why a wolf would attack a beaver that was in the water but headed for land (e.g. in a feeding canal), or conversely, wait for a beaver on land to return to water before attacking it. Nonetheless, based on our observations it appears that wolves do attack beavers in the water, pull them out of the water, and then kill and consume them on shore.

Although wolves appear to ambush beavers as a hunting strategy, there is undoubtedly a certain level of opportunism that exists when wolves are traveling across the landscape [48]. However, we do not know whether wolves first detect beavers they kill opportunistically via scent, sound or visual observation, and this could be challenging to determine. Furthermore, without direct observation or more detailed data on behavior via fine-scale movement and activity data, we cannot say whether wolves waited for, searched for, or encountered beavers opportunistically at most kill sites because we do not know how long wolves were near kill sites prior to killing beavers.

### The Key to Understanding Wolf-Beaver Dynamics?

We have provided the most thorough description of how and where wolves hunt beavers. However, there is still much to be learned about how wolves hunt beavers, and how wolf predation impacts beaver populations. To date, the impact of wolf predation on beaver populations has been estimated by: 1) calculating predation rate based on the wolf population, the beaver population, and the percent diet that is beaver (derived from scat analysis; [12]), 2) assuming a causal relationship between wolf removal and increases in beaver density [49], and 3) estimating the maximum possible predation rate for a growing beaver population [37]. By identifying kill sites, it is possible to calculate more accurate estimates of predation rates because most, if not all, of the beaver kills made by an individual wolf could be found. Other aspects of wolf-beaver dynamics could also be examined such as the impact of wolf predation on the demographic structure of beaver populations. Thus, identifying kill sites might be the key to fully understanding this important, but poorly understood, predator-prey relationship in boreal ecosystems.

## Supporting Information

S1 Appendix Individual Descriptions of Kill Sites. (PDF)

S1 Map Locations of Individual Kill Sites. (KMZ)

## References

[1] Peterson RO, Ciucci P. The wolf as a carnivore In: Mech LD, Boitani L, editors. Wolves: behavior, ecology, and conservation. Chicago: University of Chicago Press 2003 pp. 104–130.

[2] Tremblay JP, Jolicoeur H, Lemieux R. Summer food habits of gray wolves in the boreal forest of the Lac Jacques-Cartier highlands, Québec. Alces 2001;37: 1–12.

[3] Darimont CT, Reimchen TE. Intra-hair stable isotope analysis implies seasonal shift to salmon in gray wolf diet. Can J Zoo. 2002;80: 1638–42.

[4] Wiebe N, Samelius G, Alisauskas RT, Bantle JL, Bergman C, Carle R, et al Foraging behaviours and diets of wolves in the Queen Maud Gulf Bird Sanctuary, Nunavut, Canada. Arctic. 2009;62: 399–404.

[5] Nichols TC. Cooperative hunting of Canada geese (*Branta canadensis*) by gray wolves (*Canis lupus*) in northern Quebec. Can Field Nat. 2015;129: 290–92.

[6] Hall AM. Ecology of beaver and selection of prey by wolves in central Ontario. M.Sc. Thesis, University of Toronto. 1971.

[7] Baker BW, Hill EP. Beaver (*Castor canadensis*) In: Feldhammer G, Thomspon B, and Chapman J, editors. Wild mammals of North America biology, management, and conservation. Baltimore:John Hopkins University Press; 2014 pp. 288–310.

[8] Shelton PC. Ecological studies of beavers, wolves, and moose in Isle Royale National Park, Michigan. Ph.D. Dissertation, Purdue University. 1966. Available: http://docs.lib.purdue.edu/dissertations/AAI6613260/.

[9] Aleksiuk M, Cowan I. Aspects of seasonal energy expenditure in the beaver (*Castor canadensis* Kuhl) at the northern limit of its distribution. Can J Zoo.1969;47: 471–81.

[10] Slough BG. Beaver food cache structure and utilization. J Wildl Manage. 1978;42: 644.

[11] Fuller TK. Population dynamics of wolves in North-central Minnesota. Wildl Monogr. 1989;105: 3–41.

[12] Romanski MC. Estimates of abundance and predation—the population ecology of beaver in Isle Royale National Park. M.Sc. Thesis, Michigan Technological University, 2010. Available: https://irma.nps.gov/DataStore/DownloadFile/427062.

[13] Latham ADM, Latham MC, Knopff KH, Hebblewhite M, Boutin S. Wolves, white-tailed deer, and beaver: implications of seasonal prey switching for woodland caribou declines. Ecography. 2013;36: 1276–90.

[14] Peterson RO. Wolf ecology and prey relationships on Isle Royale. National Park Service Scientific Monograph Series. 11:1–210. 1977.

[15] Messier F, Crête M. Moose-wolf dynamics and the natural regulation of moose populations. Oecologia. 1985;65: 503–12. 10.1007/BF00379664 28311857

[16] Peterson RO, Page RE. The rise and fall of Isle Royale wolves, 1975–1986. J Mamm. 1988;69: 89–99.

[17] Forbes GJ, Theberge JB. Response by wolves to prey variation in central Ontario. Can J Zoo. 1996;74: 1511–20.

[18] Voigt DR, Kolenosky GB, Pimlott DH. Changes in summer foods of wolves in central Ontario. J Wildl Manage. 1976;40: 663–68.

[19] Mech LD, Smith DW, MacNulty DR. Wolves on the hunt: the behavior of wolves hunting wild prey. Chicago: University of Chicago Press 2015.

[20] Zimmermann B, Wabakken P, Sand H, Pedersen HC, Liberg O. Wolf movement patterns: a key to estimation of kill rate? J Wildl Manage. 2007;71: 1177–82.

[21] Sand H, Wabakken P, Zimmermann B, Johansson Ö, Pedersen HC, Liberg O. Summer kill rates and predation pattern in a wolf–moose system: can we rely on winter estimates? Oecologia. 2008;156: 53–64. doi: 10.1007/s00442-008-0969-2 18270746 10.1007/s00442-008-0969-2

[22] Morehouse AT, Boyce MS. From venison to beef: seasonal changes in wolf diet composition in a livestock grazing landscape. Front Ecol Environ. 2011;9: 440–45.

[23] Franke A, Caelli T, Kuzyk G, Hudson RJ. Prediction of wolf (*Canis lupus*) kill-sites using hidden Markov models. Ecol Model. 2006;197: 237–46.

[24] Demma DJ, Barber-Meyer SM, Mech LD. Testing global positioning system telemetry to study wolf predation on deer fawns. J Wildl Manage. 2007;71: 2767–75.

[25] Palacios V, Mech LD. Problems with studying wolf predation on small prey in summer via global positioning system collars. Euro J Wildl Res. 2010;57: 149–56.

[26] Sand H, Zimmermann B, Wabakken P, Andrèn H, Pedersen HC. Using GPS technology and GIS cluster analyses to estimate kill rates in wolf‐ungulate ecosystems. Wildl Soc Bull. 2005;33: 914–25.

[27] Hop K, Faber-Langendoen D, Lew-Smith M, Aaseng N, Lubinski, S. Final Report, USGS-NPS vegetation mapping program, Voyageurs National Park, Minnesota. U.S. Geological Survey, La Crosse, Wisconsin. 2001.

[28] Johnston CA, Naiman RJ. The use of a geographic information system to analyze long-term landscape alteration by beaver. Landsc Ecol. 1990;4: 5–19.

[29] Johnston CA, Windels SK. Using beaver works to estimate colony activity in boreal landscapes. J Wildl Manage. 2015;79: 1072–80.

[30] Bailey RG. 1980. Description of the ecoregions of the United States. United States Department of Agriculture. Miscellaneous Publication No. 1391.

[31] Kallemeyn, LW, Holmberg KL, Perry JA, Odde BY. Aquatic synthesis for Voyageurs National Park. U.S. Geological Survey, Information and Technology Report 2003–0001. 2003.

[32] Windels SK, Olson BT. 2015 Voyageurs National Park Moose Population Survey Report. Natural Resource Data Series NPS/VOYA/NRDS—2015/971. National Park Service, Fort Collins, Colorado. 2015.

[33] Sikes RS, Gannon WL, Animal Care and Use Committee of the American Society of Mammalogists. 2016 Guidelines of the American Society of Mammalogists for the use of wild mammals in research and education. J Mamm. 2016;92: 235–53. 10.1093/jmammal/gyw078 PMC5909806 29692469

[34] ESRI. ArcMap GIS. Ver. 10.2. Environmental System Research Institute, Inc. Redlands, California. 2015.

[35] Latham ADM. Wolf ecology and caribou-primary prey-wolf spatial relationships in low productivity peatland complexes in northeastern Alberta. Ph.D. Dissertation, University of Alberta, 2009.

[36] Google. Google Earth Pro. Ver. 7.1.4. Google, Mountain View, California. 2015.

[37] Smith DW, Peterson RO. Effects of regulated lake levels on beavers in Voyageurs National Park, Minnesota. Midwest Regional Office, Omaha, Nebraska 68102. 1988.

[38] Mech LD. The wolves of Isle Royale. National Park Service Fauna Series. 7:1–210. 1966 Available: https://archive.org/details/wolvesofisleroya00royal.

[39] Webb NF, Hebblewhite M, Merrill EH. Statistical methods for identifying wolf kill sites using global positioning system locations. J Wildl Manage. 2008;72: 798–807.

[40] Barber-Meyer S, Mech LD. Gray wolf (*Canis lupus*) dyad monthly association rates by demographic group. Can Wildl Bio Manage. 2015;4: 163–68.

[41] Buech RR. Sex differences in behavior of beavers living in near-boreal lake habitat. Can J Zoo 1995;73: 2133–43.

[42] Mech LD, Peterson RO. Wolf-prey relations In: Mech LD, Boitani L, editors. Wolves: behavior, ecology, and conservation. Chicago: University of Chicago Press 2003 pp. 131–160.

[43] Thurber JM, Peterson RO. Effects of population density and pack size on the foraging ecology of gray wolves. J Mamm. 1993;74: 879–89.

[44] Haber GC. Socio-ecological dynamics of wolves and prey in a subarctic ecosystem. Ph.D. Dissertation, University of British Columbia. 1977. Available: https://open.library.ubc.ca/cIRcle/collections/ubctheses/831/items/1.0094168.

[45] Mech LD. Possible use of foresight, understanding, and planning by wolves hunting muskoxen. Arctic 2007;60: 145–49.

[46] Basey JM, Jenkins SH. Influences of predation risk and energy maximization on food selection by beavers (*Castor canadensis*). Can J Zoo. 1995;73: 2197–208.

[47] Mech LD. The wolf: the ecology and behavior of an endangered species. Garden City: The Natural History Press 1970.

[48] Huggard DJ. Prey selectivity of wolves in Banff National Park. Ph.D. Dissertation, University of British Columbia. 1991. Available: https://open.library.ubc.ca/cIRcle/collections/ubctheses/831/items/1.0098434.

[49] Potvin F, Breton L, Pilon C. Impact of an experimental wolf reduction on beaver in Papineau-Labelle Reserve, Quebec. Can J Zoo 1992;70: 180–83.

