# Supplementary material for: Where and How Wolves (Canis lupus) Kill Beavers (Castor canadensis)
Source: PLoS One. 2016 Dec 19;11(12):e0165537. doi: 10.1371/journal.pone.0165537 (PMC5167233; doi:10.1371/journal.pone.0165537)
Supplement: S1 Appendix — (PDF) [file pone.0165537.s001.pdf]

S1 Appendix. Individual Descriptions of Kill Sites.

Below are descriptions of 22 beaver kill sites that we documented from April 2015 to November 2015 in Voyageurs National Park, Minnesota. When describing a kill site we frequently mention the location of beaver remains, or of the collared wolf as being in the vicinity of the kill site. We considered an object in the vicinity of the kill site if it was <200 m from the kill site but not at the kill site. We use the term consumption site to denote the location where a wolf consumed the beaver. In most cases the consumption occurred at the kill site. However, there were a few instances where multiple wolves were present and several consumption sites radiated out from the actual kill site (e.g., Beaver Kill Site #17 and 22). With many of the individual descriptions we have attempted to infer how the beaver was killed based on evidence at the kill site, and wolf behavior at other clusters we examined in similar locations where we did not find kill sites. In some instances there simply was not enough evidence to determine how the wolf or wolves killed the beaver, and any attempts to describe how the beaver was killed are purely speculative.

Descriptions of Individual Kill Sites

Beaver Kill Site #1 (UTM 501767, 5361064)

Kill Site Type: Below Dam

On 27 April 2015 we documented a beaver kill site from wolf V009 of the Ash River Pack. The wolf spent a minimum of 20 hr (4-hr fix interval) in a grassy area downstream from a beaver dam during which at some point it killed a beaver. There were many beaver trails going over the dam and 1 went into a small stagnant channel

(approximately 1–1.5 m wide) below the dam that connected to an old beaver pond. We found 4 wolf beds in this cluster within 3 m of each other. Given the amount of time spent at this cluster, we are unsure if these 4 beds are from a single wolf or multiple wolves. The beds were 15–20 m from the dam. In 2 of the wolf beds we found tufts of beaver hair and bone fragments. Beaver hair, dried stomach contents, and beaver bones in a small trampled area about 10 m from the beds suggested this was where the beaver was killed and consumed. This site was 15.0 m downstream from the dam. Trampled vegetation next to the stagnant channel suggested the wolf pulled the beaver out the channel and then consumed it 10 m away.

Beaver Kill Site #2 (UTM 505335, 5361076)

Kill Site Type: Below Dam

On 28 April 2015 we documented a beaver kill site from wolf V009 of the Ash River Pack. The kill occurred below a large beaver dam. There were well-worn trails and fresh cuttings on the downstream side of the dam suggesting that beavers used this area frequently. Although the kill site was close to a well-worn feeding trail that crossed over the dam, we considered this to be a below dam kill site because it was <10 m from a large dam. At the kill site we found a large area of depressed vegetation with small tufts of beaver fur, a few dried pieces of stomach contents, and a few bone fragments. The wolf appeared to have been alone and spent a minimum of 8 hr (4-hr fix interval) at this kill site. We postulate that the beaver crossed over the dam unaware of a wolf waiting below the dam, and once on the downslope of the dam was attacked immediately.

Beaver Kill Site #3 (UTM 505477, 5363871)

Kill Site Type: Near Shore

On 30 April 2015, we documented a kill site from wolf V009 of the Ash River Pack 27.2 m from the shoreline at the south end of Mud Bay, Lake Kabetogama. The kill site was easily identified as there were large amounts of beaver fur present as well as a large area of matted vegetation. A wolf scat was also present at the kill site. The wolf was probably alone at this kill and stayed there a minimum of 12 hr (4-hr fix interval).

The kill site was in a large open grassy area with the nearest woody vegetation approximately 50 m further inland. There were no beaver trails nearby and there was no evidence of fresh cuttings in the vicinity. The beaver was likely associated with an active lodge that was about 100 m away. We are uncertain how the beaver got from the water to the kill site.

Beaver Kill Site #4 (UTM 505642, 5363788)

Kill Site Type: Near Shore

We documented Beaver Kill Site #4 on 30 April 2015 100 m from Beaver Kill Site #3. This kill site was found opportunistically and based on GPS-location data wolf V009 was not near this site on the date this kill occurred. Thus, the kill was made by another wolf in the Ash River pack. Beaver Kill Site #4 was undoubtedly a different kill site as it was 1–2 days old (based on the fresh wolf scat as well a the bright red, fresh tissue on skull fragments) while Beaver Kill Site #3 was 17 days old (based on GPS-location data).

Without a GPS-collared wolf at the kill site we cannot determine how long the wolf was present at the kill site but it appears to have been alone. The only remains at the kill site were skull fragments including the incisors, a piece of the lower mandible, and the upper molariform teeth. There was depressed vegetation at the site but as was the case

with Beaver Kill Site #3, we are uncertain what occurred here. The kill site was 16 m from a small channel at the end of the bay that had < 0.3 m of water in it. There were no fresh cuttings or beaver trails nearby, and we are not sure why the beaver was at this area.

Beaver Kill Site #5 (UTM 504262, 5363766)

Kill Site Type: At Lodge

On 30 April 2015 we documented a kill site approximately 10 m from a beaver lodge on the west side of Daly Bay in Lake Kabetogama, VNP. The lodge was situated on a 10 m wide channel in tall cattails (*Typha* spp.) and canary grass (*Phalaris arundinacea*). The water level was very low and the entire lodge was out of the water. The channel was mostly dry with some water (about 1.0 m wide by 0.5 m deep) remaining. To access open water, beavers would have to travel 50–70 m via the channel.

Numerous wolf tracks surrounded the lodge. There were also wolf tracks in the muddy canal entrances to the lodge where a wolf was presumably inspecting the lodge. Wolf V009 of the Ash River pack appeared to be alone and spent a minimum of 8 hr (4-hr fix interval) at this kill site. Based on depressed vegetation, it appeared wolf V009 made contact with the beaver 1 m from the lodge and then dragged it back behind the lodge 10 m where it then consumed the beaver. This kill site was especially obvious because the dead cattails and canary grass were very brittle and broke easily with contact.

The intact beaver skull, tufts of fur, and bone fragments were at the kill site. We think the wolf waited for the beaver to exit the lodge and then attacked it. We saw no evidence in the muddy shoreline that the beaver tried to run back into the lodge with the wolf in pursuit. Therefore, the beaver had to have been leaving the lodge when attacked.

Beaver Kill Site #6 (UTM 523575, 5359361)

Kill Site Type: Near Shore

On 3 May 2015, we documented a kill site on the west shore of Little Johnson River about 1 km north of Little Johnson Lake. The kill was 16.0 m from water and wolf V028 of the Moose River pack spent a minimum of 6 hr (6-hr fix interval) in the vicinity of the kill. The wolf was likely by itself at the kill.

Like other on shore kill sites, we do not know what occurred here. The kill site was in an open grassy area next to the river with no beaver trails or active cuttings nearby. We found the skull, lower mandible, bone fragments and fur at the kill site. This beaver was likely associated with an active lodge ~150 m away. The colony had dammed the Little Johnson River about 50 m upstream of the kill site. There were no drag marks or depressed vegetation nearby.

Beaver Kill Site #7 (UTM 518406, 5360343)

Kill Site Type: Feeding Trail

On 5 May 2015, we documented a kill site on the south shore of Moose River approximately 1.5 km upstream from where Moose River flows into Moose Bay, Namakan Lake. Wolf V027 spent a minimum of 12 hr (6-hr fix interval) in the vicinity of this kill site, and was probably alone.

The kill site was on a feeding trail by an active beaver lodge situated on Moose River where beavers were cutting small aspen saplings. The kill occurred 17.7 m from Moose River. At the kill site we only found beaver fur and stomach contents. We did find tufts of beaver fur up to 1 m high in the vegetation from when the wolf was handling the beaver at the kill site.

Wolf tracks on exposed muddy shoreline of Moose River provided helpful information in understanding what occurred at this kill. We located a wolf bed close to an active beaver lodge on Moose River as well as tracks on the shoreline from the lodge to the feeding trail (distance from lodge to feeding trail was about 30 m) where the wolf tracks stopped and turned up the trail. We found tufts of beaver hair every few meters along the feeding trail until we reached the kill site. We concluded the wolf was waiting nearby when a beaver left the lodge and went ashore on this feeding trail. The wolf followed, cut off the beaver's access to water, and killed it. The tufts of fur along the trail are probably from the wolf dragging or chasing the struggling beaver up the trail.

Beaver Kill Site #8 (UTM 504617, 5360090)

Kill Site Type: Feeding Trail

On 8 May 2015 we documented a kill site from wolf V009 of the Ash River pack on the west side of the west fork of Daley Bay. Wolf V009 had spent a minimum of 9.0 hr at the kill site (the collar recorded locations at a 4 and then 5 hr fix interval). The kill site was 98.9 m from water and in a dense coniferous stand with a few deciduous trees scattered throughout. Close by were many feeding trails leading inland and the beavers appeared to be actively cutting >50 m from water in several places. The kill site was inconspicuous as it was in a coniferous stand and thus disturbances from the kill were not easily identified. However, after searching extensively we found the kill site. The only remains were long bones with some flesh still attached. We found a few fresh wolf scats and some more bone fragments in the vicinity. Based on the number of scats and scattered remains as well as several sets of wolf tracks on the shoreline, we do not think wolf V009 was alone at this kill site.

Determining what occurred at this kill site was challenging, and it is puzzling how far from water this kill occurred. With so many feeding trails nearby, it seems likely that the beaver was foraging far from water when either the beaver detected wolves, or was attacked by wolves. However, there were no fresh cuttings within 40 m of the kill site. With multiple wolves present, the beaver likely had no way to reach the water. Perhaps the beaver tried to escape by running the opposite direction of the wolves which would explain why the kill occurred even farther inland than the farthest freshly cut tree. We did not find any evidence of drag marks that would suggest the beaver was dragged to the kill site. Moreover, we thoroughly searched the entire area and ruled out the possibility that the beaver was killed closer to water and then consumed where we found the remains.

Beaver Kill Site #9 (UTM 507859, 5364552)

Kill Site Type: Below Dam

On 12 May 2015, we documented a kill site near a group of 3 ponds about 1.5 km west of Blind Ash Bay, Lake Kabetogama. Wolf V009 of the Ash River Pack was here for a minimum of 6 hr. Unlike at all the other kill sites, we found no remains at this kill. Between when the kill would have occurred and when we were able to examine the cluster we received heavy rain which certainly could have washed tufts of fur or little pieces of stomach contents to the bottom of the depressed vegetation. Similarly, it is not unreasonable that the wolves consumed the entire carcass. For example, at a few kill sites we have not found any hair and at others only hair. Therefore it is likely that either all the remains were consumed, or the few that were not were washed away and not found.

However, several other lines of evidence suggest this was in fact a kill site. We found 3 viscous, tar-like scats indicative of a recent kill (Peterson and Ciucci 2003) that

all contained beaver hair. Moreover, at the GPS-locations we found a large area that had been trampled and all the vegetation depressed. This area was significantly larger than a wolf bed and was of the same size and shape as that of Beaver Kill Sites #2 and #13. We have examined >150 clusters and have not documented depressed vegetation such as this except at other beaver kill sites.

The location of this kill fits the pattern seen at other below dam kill sites. The matted vegetation suggests the beaver was in the small channel below the dam when it was attacked and then consumed nearby on shore. Wolf V009 appeared to have been with at least 1 other wolf at this kill site based on the number of beds present. Over the course of the summer, wolf V009 visited this area frequently and we determined that in July 2015 wolf V009 killed a deer fawn and a great blue heron (*Ardea herodias*) chick <50 m from the location of this kill site. Similarly, wolf V009 killed a beaver (Beaver Kill Site #14) at the same pond as this kill site in October.

Beaver Kill Site #10 (UTM 508927, 5361953)

Kill Site Type: Small Waterway

On 15 May 2015, we documented a kill site in the middle of the Ash River territory near a small creek that was flowing into an active beaver pond. Wolf V009 appeared to be alone when attacking and killing this beaver. Based on drag marks from the creek to the kill site, it appears that wolf V009 attacked the beaver in the water and then consumed it 8.2 m away. Moreover, there was a fresh wolf track in the mud of the creek right where it appeared the beaver was attacked. The track was pointed the direction we would have expected if the wolf attacked the beaver and tried to drag it out

of the creek by backing up. The creek was narrow and shallow with most of the creek <0.5 m deep.

We found a few tufts of beaver fur at the kill site and on vegetation between the kill site and the creek presumably from when the wolf was dragging the beaver to the kill site. Nearby we found a wolf scat. Wolf V009 was at the kill site for a minimum of 4 hr (4-hr fix interval). As we searched the area we identified a rudimentary dam about 25 m upstream from the kill site. Close by were several freshly cut aspens that were likely being used to construct a dam and thus stop the flow of the creek. This dam was approximately 250 m upstream from an active beaver pond that the creek flowed into. We concluded that the beaver was likely a dispersing beaver that was trying to dam up the stream and create a pond when it was killed.

Beaver Kill Site #11 (UTM 521515, 5359451)

Kill Site Type: Small Waterway

On 2 June 2015, we documented a kill from wolf V027 from the Moose River Pack. The kill occurred about 150 m south of Wiyapka Lake next to a small waterway that went through an alder swamp. The small waterway flowed into Wiyapka Lake from a beaver pond complex 300–400 m upstream. The kill site was easily identified by the trampled vegetation, presence of stomach contents, and a large amount of beaver hair. There was a foul odor from the remains (this is the only kill site where that was the case).

We do not know how long wolf V027 was in the area because there was only 1 location taken within 200 m of the kill site. However, the maximum time spent at the kill site was 8 hr (4-hr fix interval). Technically, this kill site did not occur at a cluster as

there were no other locations prior to, or after this location within 200 m. We only visited this location because it was between 2 clusters we had to investigate. The kill site was within 5 m of the GPS location. It is worth noting that the wolf spent at least 12 hr only 225-250 m west of the kill site prior to being located at the kill site.

Based on the depressed vegetation and proximity of the kill to water, it appeared the wolf attacked the beaver in the small waterway and then consumed it 1 m away on land. There were no fresh cuttings or beaver trails nearby to suggest the beaver was on land when caught. The small channel that the beaver was pulled from was about 2–3 m wide and >1 m deep.

Beaver Kill Site #12 (UTM 515479, 5346542)

Kill Site Type: Forest Interior

On 6 June 2015, we documented a kill from V026 of the Sheep Ranch Pack. Wolf V026 was present at the kill site for at least 12 hr (12-hr fix interval). The kill site was approximately 1 km southwest of Corner Lake in a dense aspen stand 222.1 m away from the nearest body of water. We did not find any evidence of fresh cuttings or any other beaver activity. Therefore, we presumed this was a dispersing beaver that was trying to move from 1 pond complex to the next by crossing through the forest. Wolf V026 had ventured 1 km south of the Sheep Ranch Pack territory on the same day and likely encountered the beaver opportunistically.

At the kill site we found evidence of a struggle as a downed log had been torn apart on 1 end with claw and/or tooth marks present in the wood. A small sapling had also been broken off about 1 m above the ground, and we found beaver fur on the sapling where it had been broken off. More beaver fur was found in the vegetation nearby, and at

the kill site. We also found a wolf scat at the kill site. About 15 m away from the kill site we found some bone fragments that were in the same spot as the second GPS location from wolf V026. It appeared that wolf V026 was alone at the kill site as all beaver remains that were identified were found at the GPS locations from that wolf.

Beaver Kill Site #13 (UTM 511374, 5363576)

Kill Site Type: Below Dam

On 10 October 2015, we documented a kill site 75 m west of the Sullivan Bay trailhead below a dam near an active pond. The kill occurred below the 2nd active dam downstream of the pond instead of the primary dam that was damming the main pond. The 2 dams were about 30 m apart, and a well-used beaver trail crossed over the primary dam, across a small stretch of land and into the small pond created by the 2nd dam. There was also a trail that crossed over the 2nd dam to a small shallow channel below the dam. The kill site was about 3 m from the trail and 10 m below the second dam (distance to the small channel was 4 m). We speculate that the beaver had crossed over the second dam and had almost reached the small channel when a wolf, concealed in the vegetation, attacked the beaver and consumed it close by.

The kill site was obvious due to the large area of trampled vegetation but we found very little of the carcass at this location (a tuft of beaver fur and stomach contents). Wolf V009 of the Ash River pack was present in the vicinity of the kill site for at least 24 hr (6-hr fix interval). However, between 6 and 12 hr the wolf moved 140 m away where he bedded down for at least another 18 hr on a high rocky ridge. We found 3 fresh wolf scats on this ridge – all were associated with GPS-locations. Similarly, we found the

beaver skull close by 1 of the GPS-locations. Based on this V009 was likely alone at the kill site.

Beaver Kill Site #14 (UTM 507558, 5364569)

Kill Site Type: Feeding Trail

On 13 October 2015, we opportunistically documented a kill site on the southwest side of the beaver pond where Beaver Kill Site #9 was found. Although we found this kill site opportunistically, we think that wolf V009 killed this beaver because GPS-collar data (6-hr fix interval) shows that he was 170 m from the kill site about 2 hr after we found it. We do not think it is coincidence that the wolf was within the vicinity of this kill site just after we documented a freshly-killed beaver.

The kill occurred on an active feeding trail where beavers had been cutting and transporting aspen to the pond. Wet blood across the ground and vegetation suggested the kill site was < 12 hr old. Rain throughout the previous day would have washed away the blood if the kill had occurred before or during this time.

At the kill site we found intact segments of the intestinal tract and fresh pieces of muscle tissue that had not been consumed. We probably scared the wolf away from the carcass as we did not find these remains at any other kill site. We also found a piece of leg bone still attached in the socket to a piece of the pelvis. Some tufts of beaver fur were spread around the kill site and stomach contents had been strewn about. The wolf appeared to be alone as all beaver remains found were at the kill site. We searched the area extensively and did not find any beaver remains or beds in the vicinity of the kill. We do not know how long Wolf V009 remained or would have remained at the kill site had we not scared it away.

On the shore of the pond we found a set of fresh wolf tracks in the mud. The tracks were on the west side of the feeding trail and headed east along the pond shore. We did not find any tracks in the stretches of exposed muddy shoreline on the east side of the feeding trail leading us to believe the wolf stopped traveling on the shore when it reached the feeding trail. This seems likely and is consistent with how wolf V027 hunted and killed a beaver on a feeding trail at Beaver Kill Site #7. We do not know whether this wolf was waiting until the beaver went on shore, or was running the shoreline when it caught the fresh scent of a beaver, turned onto the feeding trail, and found the beaver inland. Whatever the case, the beaver appeared to be cutting a recently-felled aspen when, based on the drag marks and blood on the vegetation, it was attacked and then consumed about 10 m away. The kill site was 15.5 m from the pond.

Beaver Kill Site #15 (UTM 505693, 5360680)

Kill Site Type: Feeding Canal

On 13 October 2015, we documented a kill site on the south shore of the east fork of Daley Bay, Lake Kabetogama. Wolf V009 of the Ash River Pack was present at this kill site for at least 6 hr (6-hr fix interval) and appeared to be alone. The dead beaver was from the same lodge as the beaver found at Beaver Kill Site #16.

This kill site was 4.9 m from an active feeding canal that was about 30–35 m long, 1 m wide, and about 1 m deep. Depressed vegetation from the shore of the canal to the consumption site suggests the wolf attacked the beaver in the water and then consumed it away from the canal. We only found a few small tufts of beaver fur, stomach contents, a few bone fragments, and 2 castor glands. We did not find any fresh cuttings or other beaver activity at the kill site. Thus, there is no reason to believe the beaver was on

shore when attacked. The beaver was likely pulled out of the water about half way down the feeding canal. There was no evidence (depressed vegetation or tracks) on the shoreline to suggest the wolf attempted to pull the beaver out of the canal at any other spot.

Thus, we think the beaver was heading toward the feeding trail at the end of the feeding canal and wolf V009 was waiting in the tall grass on the canal edge. Wolf V009 then attacked and killed the beaver. The depressed and trampled grass at the kill site made it easy to identify. However, very little of the beaver was left.

Beaver Kill Site #16 (UTM 505770, 5360683)

Kill Site Type: Feeding Canal

On 13 October 2015, we documented a kill site on the north shore of the east fork of Daley Bay. The kill occurred near Beaver Kill Site #15 and the beaver killed at this kill site (#16) belonged to the same lodge as the beaver at Beaver Kill Site #15. Wolf V009 of the Ash River pack spent at least 30 hr (6-hr fix interval) at this kill site. We believe multiple wolves were present because we found several scats, and consumption sites within 50 m of the kill site.

The beaver appeared to have been pulled out of a wide feeding canal, dragged on shore, and consumed 2.6 m from water. The canal was not a narrow, long canal like at Beaver Kill Site #15 but rather was short and funnel shaped as the mouth of the canal was quite wide but then narrowed to about 1 m when it met the shore. Given the shape of the canal and angle of the shoreline, the canal was likely >1 m in most places.

The beaver was attacked 3 m from the end of the canal. Wolf V009 or another wolf it was with were likely waiting on the canal edge and attacked the beaver as it was

heading for the feeding trail at the end of the canal. The beavers had been especially active at this feeding trail and had likely cut more than half of the trees that were <30 m from shore.

We left this kill site at dusk and howled to elicit a wolf response. Within seconds the entire Ash River Pack (4 adults, 2 pups) howled back to the northeast of the kill site. The 2 pups in the pack could be heard clearly, and we estimated that the wolves were no farther 300 m away.

Wolf V009 first arrived in the vicinity of the kill site on 11 October 2015 and the last location at the kill site was on 12 October 2015 at 9:30. We do not know then if it was simply coincidental that the entire pack was near this kill site at this time or if some pack members remained in the area for up to 48 hr following the kill.

Beaver Kill Site #17 (UTM 509974, 5361135)

Kill Site Type: Feeding Trail

On 26 October 2015 we documented a kill site at a pond about 1 km due west of the Helipad Road. Wolf V009 of the Ash River Pack was present at this kill site for at least 12 hr (6-hr fix interval). The kill was hard to find as the few remains present were inconspicuous. We were only able to identify a few tufts of beaver fur, stomach contents and both castor glands. However, only the few small pieces of the stomach contents were present at the actual kill site.

The week preceding this kill site was generally cold, and the ground and leaf litter were frozen as a result. Thus, the evidence of a struggle or of the handling of the beaver that is usually apparent from disturbed leaf litter or vegetation was mostly missing. Nonetheless, upon searching the area we found some small pieces of stomach contents in

a ‘bread crumb’ like trail back to the pond and were able to determine that the kill occurred 1 m off of a feeding trail and 5.1 m from the water. Some depressed vegetation was present at the kill site but it would not have been obvious without following the remains back to the location of the kill.

We determined that there were 2 wolves present at this kill based on the fact that we found 2 separate trails of beaver remains going in different directions from the kill site. It appeared as though 1 wolf dragged part of the beaver 20 m south –leaving a trail of stomach contents– to a consumption site. The other wolf dragged the rest of the beaver in an east-southeast direction about 25 m (leaving tufts of beaver fur caught in the vegetation) to a consumption site. We found 3 wolf scats in the vicinity of the kill. We did find a few wolf beds next to active beaver trails around this pond about 30–50 m from the kill site. However, we do not know whether the wolves waited for the beaver to come ashore or not.

Beaver Kill Site #18 (UTM 507510, 5367693)

Kill Site Type: At Dam

On 26 October 2015, we documented a kill site where wolf V045 of the Shoepack Lake Pack killed a kit beaver approximately 200–300 m north-northeast of the Shoepack Beach Campsite, Lake Kabetogama. Wolf V045 was present at this kill site for 7.7 hr (20-min fix interval). The kill occurred on a small point that jutted out into a pond about 1 m away from the beaver dam. Based on the GPS data, wolf V045 was present at the kill site on the small point for at least 1.3 hr and then moved 50 m away and bedded down for at least 6.3 hr. A scat was present at both the kill site and also where the wolf bedded down nearby. The scat at the kill site was full of beaver stomach contents but very little

beaver hair. The small size of the kit might have led to the wolf consuming a significant amount of the stomach contents which were then defecated. All beaver remains found were located on the point. Wolf V045 appeared to be alone at the kill site. The only remains identified were a small pile of stomach contents and 2 small incisors indicating this was a beaver kit.

The depressed vegetation on the point suggested the beaver was in the water when wolf V045 attacked it. The beaver was then killed and consumed 1 m from the water on the point. Based on the evidence, there are 3 different ways this kill could have occurred: 1) the wolf waited on the point and a beaver swam right next to shore and the wolf grabbed it without going in the water, 2) the beaver was out in the pond a few meters from shore, the wolf waited until it was close enough and then leaped into the pond and caught it, or 3) the wolf was swimming in the water, caught the beaver, and then dragged it up onto the point.

Beaver Kill Site #19 (UTM 508825, 5350058

Kill Site Type: Feeding Trail

On 30 October 2015, we documented a kill site from the Sheep Ranch Pack at a pond approximately 1 km north of the northwest corner of the Sheep Ranch. Wolf V026 was present at the kill site but we think that the wolf was with several other wolves based on the number of beds at the kill site. The beaver pond had several long narrow channels to access feeding trails on the shore. The kill occurred on a feeding trail only 8.6 m from the water. Based on the depressed vegetation, the beaver was probably on land when attacked and killed based on the depressed vegetation.

Most of the remains present at the kill site were tufts of beaver fur on the ground. However, beaver fur was also caught in briars and other small shrubs. The lack of remains was likely due to the fact that multiple wolves were present. We found 3 wolf scats nearby. It appeared the beaver was coming from the feeding canal to the feeding trail. Once it was on shore, the wolves attacked, and consumed the beaver.

Wolf V026 was present at the kill site for at least 7.7 hr (20-min fix interval). Based on the GPS data it appeared as though the wolf spent 1–1.7 hr near the pond close to the kill. If we assume that the kill occurred when the wolf was at the exact location of the kill site then we can say that the wolf was at this beaver pond 40 min prior to making or helping make the kill. However, we do not know when the kill occurred and since there were other wolves present we do not know if wolf V026 or other pack members made the kill. Wolf V026 then moved 30 m east of the kill site and bedded down for several hr on a small ridge. Other wolf beds were present on the ridge and we think that several wolves bedded down in this area after the kill.

Beaver Kill Site #20 (UTM 515020, 5366546)

Kill Site Type: Feeding Trail

On 4 November 2015, we documented a kill site on the north shore of Kohler Bay, Lake Kabetogama. Wolf V045 of the Shoepack Lake Pack was present in the vicinity of the kill site for at least 25.7 hr (20-min fix interval). The kill site was on a feeding trail 23.1 m from the water. The beavers had been very active in this area and near the kill site there were several freshly-cut aspens.

We found this kill site easily as only 85% of the beaver had been consumed. We found the skull and lower mandible as well as the entire vertebral column with the bones

of the back legs still attached. Wolf V045 was likely alone at this kill site as all beaver remains were at GPS-collar locations. Wolf V045 was initially at the kill site for at least 1.3 hr before it left for a few hours then returned and stayed for at least 24.3 hr more. The beaver was an adult based on skull size and could have weighed 18–22 kg. Therefore, it would have been a significant amount of food for a single wolf, which likely explains why the wolf was in the vicinity of the kill site for so long.

We know when this wolf arrived at the kill site but we do not know when it made the kill. All of the initial locations were <10 m apart but it is unknown whether that was the result of the wolf waiting at the kill site. We would expect the same cluster pattern from a wolf that waited and then made a kill, and from a wolf that immediately made a kill and consumed the beaver at that location.

Beaver Kill Site #21 (UTM 507733, 5364461)

Kill Site Type: Feeding Trail

On 4 November 2015, we documented a kill site at the same pond complex as Beaver Kill Site #9 and #14. Wolf V009 of the Ash River Pack was present at this kill site for at least 6 hr (6-hr fix interval). Throughout the ice-free season Wolf V009 frequently visited and spent a significant amount of time around this pond complex (V009 killed 3 beavers, 1 fawn and 1 great blue heron chick in this area).

Wolf V009 was not alone at this kill site. We found bone fragments in a few areas about 20 m from the kill site in the woods which suggests that other wolves were present. We also found several wolf beds near the kill site. One wolf scat was found approximately 30 m from the kill site.

The kill occurred on a feeding trail 12.8 m from the water. The beavers had been clearing many large aspens in the area near the kill site. We are not sure how the wolf or wolves hunted this beaver, and GPS data do not provide clarification. At the kill site we found stomach contents and tufts of beaver fur strewn across the vegetation. Some small bones and bone fragments were found at the kill site as well.

Beaver Kill Site #22 (UTM 534251, 5363143)

Kill Site Type: Feeding Trail

On 6 November 2015, we documented a kill site on the south shore of a beaver pond just north of O'Leary Lake. Wolf V033 of the Moose River Pack was present at this kill site for at least 5.3 hr (20-min fix interval). We found the kill site 13.4 m from the water on an active feeding trail. The kill was relatively fresh and we found wet blood on the leaf litter and vegetation at the kill site. The beaver appeared to have been attacked about 8 m from the pond but then dragged farther inland to where it was consumed.

We think that the entire Moose River Pack (8 adults, 3–4 pups) was present at this kill site. In the fall this pack appeared to be moving around its territory nomadically and almost every cluster we visited had several beds and scats present. Moreover, for most of the fall we had 2 GPS-collared wolves in this pack and they were almost always in the same location at the same time. We visited the clusters prior to, and after this kill and found numerous wolf beds and wolf scats at both.

At the kill site we found strewn stomach contents, a few tufts of beaver fur, and some small bone fragments. We searched the area and found several wolf beds 25-75 m from the kill site with beaver fur, beaver blood, stomach contents or bone fragments in them. Similarly we found the skull and lower mandible 180 m from the kill site in an area

where a few wolves appeared to have bedded down. Whether the wolves were searching for or waiting for this beaver is unknown. Wolf V033 was at the kill site for several hr but we do not know if the wolves had made the kill yet or if they were waiting to make the kill. In the vicinity of the kill site we documented several wolf beds next to beaver feeding trails but we cannot say whether this was from wolves waiting for a beaver to come ashore, or simply the result of them bedding down after making the kill.
